# Force-stabilizing synergies can be retained by coordinating sensory-blocked and sensory-intact digits

**DOI:** 10.1371/journal.pone.0226596

**Published:** 2019-12-17

**Authors:** Wei Zhang, Sasha Reschechtko, Barry Hahn, Cynthia Benson, Elias Youssef

**Affiliations:** 1 Department of Physical Therapy, City University of New York / College of Staten Island, Staten Island, New York, United States of America; 2 Department of Physiology and Pharmacology, University of Western Ontario, London, Ontario, Canada; 3 Emergency Medicine, Staten Island University Hospital, Staten Island, New York, United States of America; Washington University in Saint Louis School of Medicine, UNITED STATES

## Abstract

The present study examined the effects of selective digital deafferentation on the multi-finger synergies as a function of total force requirement and the number of digits involved in isometric pressing. 12 healthy adults participated in maximal and sub-maximal isometric pressing tasks with or without digital anesthesia to selective digits from the right hand. Our results indicate that selective anesthesia paradigm induces changes in both anesthetized (local) and non-anesthetized (non-local) digits’ performance, including: (1) decreased maximal force abilities in both local and non-local digits; (2) reduced force share during multi-finger tasks from non-local but not local digits; (3) decreased force error-making; and (4) marginally increased motor synergies. These results reinforce the contribution of somatosensory feedback in the process of maximal voluntary contraction force, motor performance, and indicate that somatosensation may play a role in optimizing secondary goals during isometric force production rather than ensuring task performance.

## Introduction

The human hand is a redundant motor system [1] because it has more degrees of freedom than necessary to perform most manual actions [2–5]. For example, while drinking water from a glass, the lifting force distribution among individual digits is undefined since there are an infinite set of lifting forces which could be applied by individual digits to equal the weight of the glass. Coordinating the individual fingers of the hand could pose a control problem in terms of choosing some specific combination of finger forces to satisfy task constraints (like lifting the glass); one proposed solution is the notion of motor synergies [6–7]. In this parlance, motor synergies are defined as neural organizations representing co-variation of elements which can be individually controlled (“elemental variables”) in order to stabilize the goal behavior (“performance variable”) or some other behavior(s) considered to be important by central nervous system in a specific motor task. In the multi-finger force production scenario, for an example, element variables refer to individual force produced by each digit while performance variable is the resultant force by all digits. Multi-finger synergies have been investigated within the framework of the uncontrolled manifold (UCM) hypothesis [8–9] in a number of studies involving a variety of manual tasks underlying both healthy and pathological conditions [10]. In these studies, synergies are typically quantified based on the structure of variability in the space of elemental variables, which is decomposed into two subspaces: (1) UCM and (2) its complementary (ORT) subspace. The proportion of variability within the UCM subspace has been called ‘good’ since this variance does not affect the performance of the task. Correspondingly, the component of variability in ORT subspace is sometimes called ‘bad’ because variance within this subspace introduces changes in the performance variable [11–12]. Synergies can thus be assessed as a result of relative comparison between the two variance components, ‘good’ versus ‘bad’, in the space of elemental variables [11,13–14]. Despite extensive study of synergies within the UCM framework, much less is known about the extent to which sensory feedback contributes toward this implementation of multi-element coordination.

Multi-finger synergies can be changed by limiting access to different sensory modalities. For example, coordination across digits is influenced by varied visual feedback conditions [3,15] or becomes weaker when the palmar area of the hand is vibrated (presumably due to changes in proprioceptive acuity as a result of this stimulation) [16]. In recent studies, a nerve block procedure at the digit or wrist levels has been used to investigate synergies underlying a deafferented hand model [14,17]. However, no consensus has been found regarding the extent to which the somatosensory information affects multi-finger synergies during accurate hand motor control. Motor coordination in a redundant system likely results from both feed-forward [18] and feedback processes [19–20]. As such, determining how somatosensory information contributes to the multi-element motor coordination requires further study.

A potential confound in using a deafferented hand model to study how somatosensory information affects inter-finger coordination lies in the notion of signal-dependent motor noise [21–26]. A number of studies have shown increased variability in force production as the magnitude of force production increases; crucially, however, anesthesia decreases force production ability. Deafferentation induced by local anesthesia at digit [27–29] or wrist levels [30–31] has been reported to result in altered force sharing patterns [14,32], weakened digital force covariation [14,17], and disturbed digital force synchronization [33]. However, because digital anesthesia reduces maximal force ability [14,30,32,34–36], it is important to determine whether the aforementioned changes in structure of motor variability is resulting directly from the absence of somatosensory information, or simply from decreased force production during digital anesthesia.

In the current study, we address these issues by using a previously developed deafferented hand model [14,32]–using digital anesthesia on selective digits of the test hand–and combining this with tasks evaluating force production at varied force levels. We asked subjects to perform a series of isometric force production tasks and investigated (1) the effect of deafferentation of selective digits on the force-stabilizing multi-finger synergies, and (2) the effect of force magnitude levels on synergy strength under digital anesthesia and with intact sensation. We hypothesized that selective digital deafferentation would lower force-stabilizing synergies, and that this difference will decrease with lower forces and fewer fingers explicitly involved in the task.

## Methods

### Subjects

A total of 12 healthy adults (six males and six females; age: 25.6 ± 4.1 years old [mean ± standard deviation]; weight: 81.3 ± 14.6 kg; height: 172.6 ± 10.1 cm) participated as subjects in the current study. All subjects were right-handed and given an Edinburgh Handedness Inventory score of 100. No subject reported any history of neurological, musculoskeletal, vascular, metabolic disorders, and/or upper limb impairments, and none reported allergies to the anesthetic agents or preparation materials. Subjects were unaware of the research-expected results and gave written informed consent in accordance with the Declaration of Helsinki. The current research protocol was approved by the Institutional Review Board at the City University of New York and Northwell Health.

### Apparatus

A customized isometric-force testing system was used in the current study. All subjects performed pressing isometric force production by four fingers of the right hand during the experiment. We used four Nano-17 force/torque (F/T) transducers (ATI Industrial Automation Inc, Apex, NC) to measure the individual force produced by each finger: (1) Index (I); (2) Middle (M); (3) Ring (R); and (4) Little (L). Sensors were covered with 100-grit sandpaper in order to prevent finger slippage. An acrylic glass plate with four slots (2.5 cm center to center) was fixed on the table to provide a mounting base for the sensors. Each sensor fit into one slot. Before the experiment, all four sensors were moved within the slots distally/proximally in order to accommodate a subject’s individual hand shape and finger lengths. Force data were sampled at 1000 Hz and digitized using a 16-Bit analog-to-digital board (PCI-6225; National Instruments, Austin, TX). A customized program written in the National Instruments LabVIEW computing environment logged data for offline processing and displayed real-time feedback to the subjects.

### Experimental procedures

**Fig 1:**
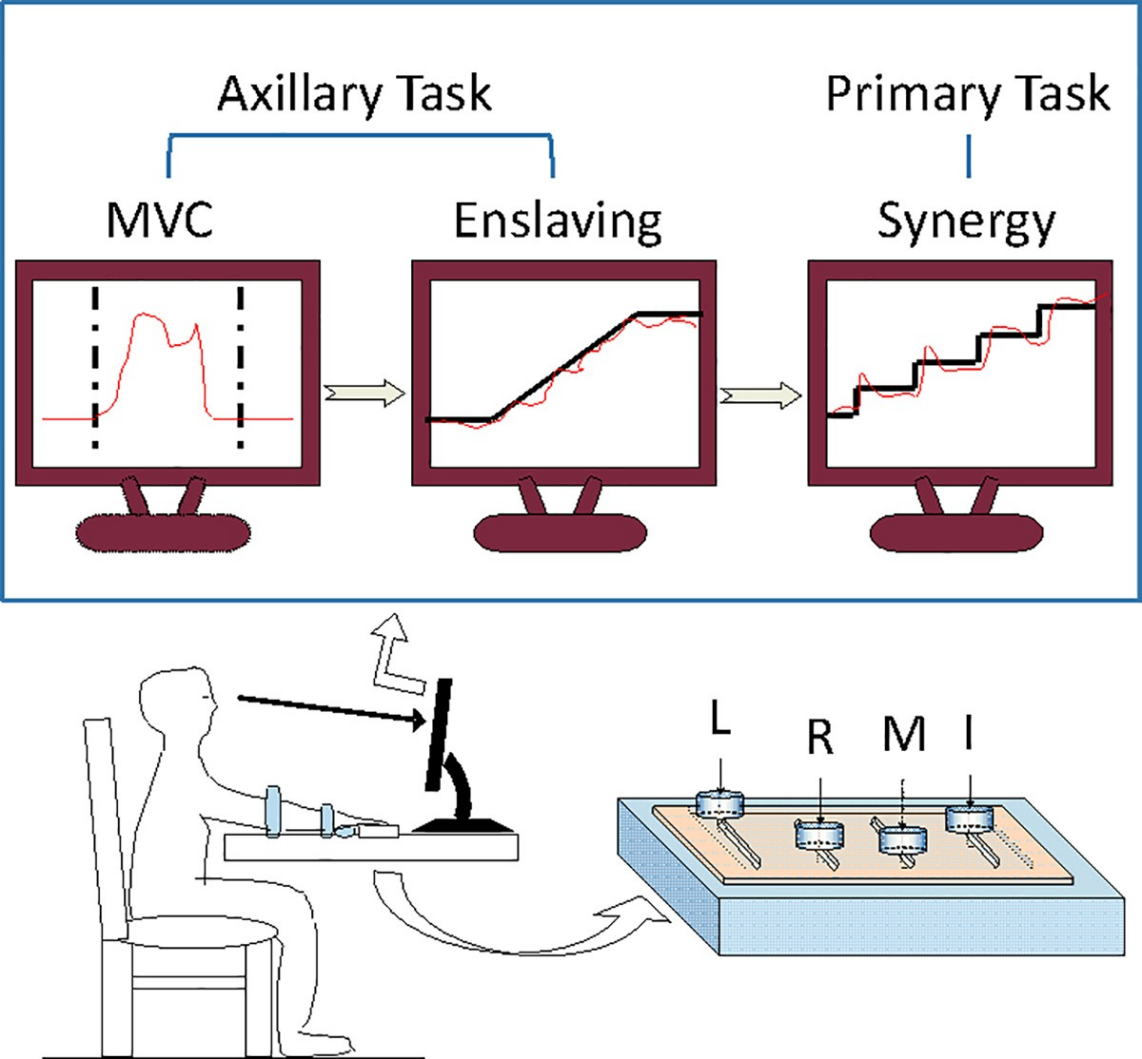
Experimental set up. Side view of a subject sitting in front the experimental set up is showed. Four force/torque sensors (ATI nano17) are mounted on the top of a table to measure subjects’ force produced by each individual finger: index (I), middle (M), ring (R) and little (L). Both task time templates and subjects’ instant force production are displayed on a LED screen for subjects over each trial. There are three isometric force production tasks including two axillary tasks (MVC task and Enslaving task) and one primary task (Synergy task). Subjects are instructed to utilize either individual finger or finger combination to perform these tasks.

In order to prepare for the procedure, subjects were asked to sit in front of the customized experimental set up mentioned above and face a 24” computer screen (Fig 1). A subject rested his/her right forearm horizontally in a U-shape polyethylene tube, padded with sponge to provide comfort, in a palm down position. During the experiment, the subject’s right forearm was immobilized by two hook-and-loop straps inside the tube in order to maintain 45° elbow and shoulder flexions. Before each individual experimental trial, subjects were instructed to rest their finger pads area on corresponding F/T sensor and their rest palm areas on a shape-customized clay block in order to maintain 30° of flexion at metacarpophalangeal joints and less than 20° of flexion at interphalangeal joints.

For each subject, there were three isometric force production tasks, including two ancillary tasks: maximal voluntary contraction (MVC) and enslaving task; and one primary task: synergy task (Fig 1). The MVC task was used to evaluate fingers’ maximal force abilities via maximal voluntary contraction by each of four individual fingers, i.e., index only (I), middle only (M), ring only (R), and little only (L), as well as by all four fingers together (IMRL). During each MVC trial, subjects were encouraged to press as hard as possible by using the designated finger or finger combinations on the corresponding sensor(s) within a six-second time window after a verbal ‘go’ signal. The subject’s total force production was displayed online as a time-course yellow-cursor template on a computer screen over each trial course. Two trials were performed for each digit condition, and the trial with larger maximal force was chosen to be analyzed in the study. Results of maximal forces measured in individual-finger MVC tasks were applied to pre-determine the target forces in the enslaving tasks, while the four-finger MVC forces were used to specify each subject’s target forces in synergy tasks. Experimental conditions were presented in a pseudo-randomized order across subjects.

Both the enslaving and synergy tasks involved following a target force-time template displayed on a computer monitor. The enslaving task was used to determine individual finger’s independence of force production, a phenomenon in which unintended force production by non-instructed fingers occurs during instructed fingers’ force production of the same hand. The enslaving matrix [5] was constructed from the enslaving task and was used in a further analysis to quantify motor synergy (described later) from the synergy task. We adopted frequently used templates that adopt controlled and relatively low forces for individual-finger actions. During this task, subjects were instructed to press with one finger (I, M, R, or L) following a time-force template line displayed on the computer monitor. The template line had three straight line segments, which were based on the subject’s individual finger maximal force as tested in the MVC task: a 1-sec horizontal segment equal to 0% MVC followed by a 4-sec oblique segment going up from 0 to 10% MVC ramp and ending by a 1-sec horizontal segment equal to 10% MVC. Each instructed digit performed one test trial after three practice trials. It was important that all the non-instructed fingers were required to maintain contact with the corresponding sensors during the task although subjects were told not to pay attention to the possible force exerted by them. An ongoing total force produced by all fingers was also displayed as a cursor on the screen to provide instant feedback.

The primary task, the synergy task, was to investigate the multi-finger motor synergy via an uncontrolled manifold hypothesis framework (UCM) [6]. Similar to the enslaving task, the task template line in the synergy task was composed of five horizontal segments based on the subject’s four finger maximal force as tested in the MVC task, starting with 0% MVC for 1 sec followed by 3 sec of each of 2.5%, 5%, 7.5%, and 10% MVC. Based on different fingers’ involvement, two conditions were presented in a pseudo-random order across subjects, that is one condition with all four fingers tracing the target line together (IMRL) and the other condition by adding I, M, R, and L for each force level increase in a sequence (I+M+R+L), i.e., 2.5% MVC by I, 5% MVC by I and M, 7.5% MVC by I, M, and R, and 10% MVC by all four fingers. Both conditions were designed as typical isometric pressing force production tasks to evaluate multi-digit synergies [13]. The IMRL condition was to determine force-stabilizing synergies among all sensory-blocked and sensory-intact digits as a function of target force effort, whereas the I+M+R+L condition was to further investigate the alternation of the multi-finger synergies attributable to digital involvement. Subjects were asked to perform 25 trials after five practice runs. In order to prevent fatigue, we gave at least 10-sec and 5-min rest intervals between trials and among conditions and tasks, respectively. In I+M+R+L condition, all the non-instructed fingers were required to maintain contact with the corresponding sensors. However, if subject failed to follow the instruction and produced identifiable force (> 0.5 N) by any non-instructed finger, the specific trial would be omitted and redone immediately. Subjects’ forces produced by all four fingers including non-instructed fingers were reflected in the visual feedback on the computer monitor.

### Digital anesthesia

In order to evaluate the effect digital sensory feedback absence on multi-finger motor performance and motor synergy, subjects performed the above experimental procedure repeatedly in two sessions: (1) *Control* and (2) *Anesthesia*. These sessions were presented in a pseudo-random order across subjects with at least a 2-week interval in between each session. In the anesthesia session, subjects received digital anesthesia on their right-hand index and middle fingers (Staten Island University Hospital, Staten Island, NY) (see details in [14]). The locally injected anesthetic was a mixture of 1% lidocaine and 0.5% bupivacaine (50:50), and was administered at digital nerves in the web space. Up to three sets of injections could be performed per finger until the subject reported complete numbness in that specific digit. A low dosage was used for the initial injections and gradually and incrementally added in later applications as needed (not exceeding 10 ml total per subject). This was done to block sensory but not motor nerve fibers in the injected finger [14, 37]. A set of Von Frey hairs (Stoetling Co., Wood Dale, IL) was used to ensure subject’s tactile sensation was successfully blocked in the injected fingers (size 6.65, 300 g), and remained intact on non-injected fingers and rest of the hand (size 2.83, 0.07g). Subjects who received three times of injections at a particular digit and did not reach complete numbness were excluded from the data collection in the present study.

### Data analysis

Experimental variables were analyzed offline by using MATLAB (MathWorks), Excel (Miscrosoft), SPSS (IBM) and Origin (OriginLab). As we described earlier, variables quantified in the MVC and enslaving tasks were used intermediately either to establish further experimental tasks or in UCM data analysis; therefore, experimental variables in these two ancillary tasks were described but not be emphasized in our data presentation and report. In the MVC task, the maximal pressing force (F_MAX_) in I, M, R, L, or IMRL was expressed in newton. In the enslaving task, force production by the instructed finger (also called master finger, i.e., I, M, R, or L) and non-instructed fingers (also called enslaving fingers) were used to compute the *n*×*n* enslaving matrix (*E*) for the right hand, where n equals the total number of fingers involved in the task. For example, when the master finger was I, enslaving fingers were M, R, and L. Entries in *E* represented the relative amount of force change in individual finger versus the total force during single-digit force production (see details in [13,38]).

In the synergy task, in order to evaluate the force contribution from each finger toward the overall force production required by the task, the individual force (N) at each finger was averaged over the intermediate second for each 3-sec force level per trial. In addition, in order to evaluate a subjects’ task performance, we quantified the accuracy of the overall force production relative to task-required template force by calculating the root mean square error (RMSE) for all force levels. Similarly, averaged values over the intermediate sec for each 3-sec force level per trial were reported in our results. Furthermore, in order to evaluate the motor coordination among multiple fingers, such as whether the individual fingers were coordinated to stabilize the total force (F_TOT_) produced by all, we quantified the motor synergy in the framework of the UCM hypothesis. Within the UCM framework, individual finger force data (F) were converted into hypothetical commands to fingers, modes (m), as *m* = [*E*]^−1^F, in which E denotes the 4×4 enslaving matrix from right hand, which was computed from the enslaving task. Thereafter, in the mode space, the total cross-trial variance (V_TOT_) was calculated for each time sample based on 25 trials performed by each subject. V_TOT_ consists of two variance components: (1) one lies in the UCM subspace (V_UCM_) and (2) the other lies along the orthogonal to the UCM subspace (V_ORT_). The former indicates that the individual mode cross-trial variance does not affect the total performed value of F_TOT_, while the latter reflects the amount of mode variance in the collected data set that leads to changes in F_TOT_. An index ΔV was therefore used to quantify the multi-finger synergy, which was calculated as the variance difference between two components (V_UCM_ and V_ORT_) and further normalized by the total amount of variance for each time sample:

$$\Delta V=\frac{\frac{V_{UCM}}{n-1}-V_{ORT}}{\frac{V_{TOT}}{n}}$$ Eq 1

In the above equation, the total variance and its components were calculated per dimension according to the finger mode space, where the dimension of the total variance space were n and that of UCM and ORT subspaces were n-1 and one, respectively. For an example, the finger mode space is two-dimensional when I and M were involved to perform 5% MVC in I+M+R+M task condition, and n denoted in Eq 1 equals two accordingly. When ΔV > 0, more V_UCM_ (per dimension) than V_ORT_ was observed, reflecting a multi-finger synergy stabilizing F_TOT_. In contrast, ΔV = or <0 can be interpreted as an anti-synergy where individual finger forces co-vary to *change* F_TOT_ rather than *stabilize* it. Note the motor synergy was used to be quantified in a redundant system, i.e., more element variables (finger modes) than performance variable (total force). Theoretically this means that for I+M+R+M task condition, first force level at 2.5% MVC performed only by one finger (I), ΔV cannot be calculated. However, because feedback was provided on total force throughout the procedure, we analyzed all tasks in the redundant 4-dimensional space reflecting the feedback. In statistical analyses, ΔV indices were averaged over the intermediate second for each 3-s force level per subject.

### Statistical analysis

We performed multiple mixed-effect analysis of variances (ANOVAs) with repeated measures. All of the factors described below were within-subject factors. In order to evaluate the effect of selective digital anesthesia on individual and all digits’ maximal force production, a two-way ANOVA was performed on F_MAX_ with the factors of *Session* (Control versus Anesthesia) and *Cond_MVC* (I, M, R, L, and IMRL). In order to determine if the enslaving effect was altered after selective digital anesthesia, we performed a two-way ANOVA on master finger’s *E* entries when I, M, R, or L were the master finger with the factors of *Session* and *Digit* (I, M, R and L). In order to identify effect of digital anesthesia on the total force distribution among all the digits in the synergy task, we performed a three-way ANOVA on the individual finger forces (in Newton) while contributing to the highest force level (such as 10% MVC) since subjects were asked to use all four fingers during this force level in both the IMRL and I+M+R+L synergy tasks. This specific 3-way ANOVA included Session, *Cond Synergy* (IMRL versus I+M+R+L) and *Digit* factors. In order to examine the subjects’ performance of task accuracy before and after partial removal of somatosensory feedback in the hand, a three-way ANOVA was performed on RMSE in synergy task with factors of *Session*, *Force-Level* (four levels consisting of 2.5%, 5%, 7.5%, and 10%), and *Cond Synergy*. In order to investigate the absence of digital sensory feedback on multi-finger motor synergy, we performed the same 3-way ANOVA as described above on the index ΔV for IMRL and I+M+R+L synergy tasks separately with factors of *Session* and *Force-Level* (four levels for IMRL consisting of 2.5%, 5%, 7.5%, and 10% and three levels for I+M+R+L consisting of 5%, 7.5%, and 10%). The same two-way ANOVAs were also performed with the variance components: (1) V_UCM_ and (2) V_ORT_. Because synergy indices are bound according to computational limits, we applied Fisher’s z-transformation on ΔV index before performing the statistics as the follows: ΔVz = 0.5 (ln (ΔV- B_MIN_)—ln (B_MAX_ - ΔV)) where B_MIN_ and B_MAX_ are the lower and upper limits, respectively. Specifically, B_MIN_ and B_MAX_ denote -4 and 4/3 in our analyses because subjects always received feedback on total force, even when they were explicitly instructed to press with fewer than 4 fingers. When the assumption of sphericity was violated, the Greenhouse–Geisser correction of degrees of freedom was used. Post hoc tests for pairwise comparisons were performed with Bonferroni adjustments when appropriate. The level of significance was taken as p < 0.05.

## Results

All subjects successfully completed the two ancillary tasks and one primary task following instructions in both the anesthesia and control sessions.

### MVC task

**Fig 2:**
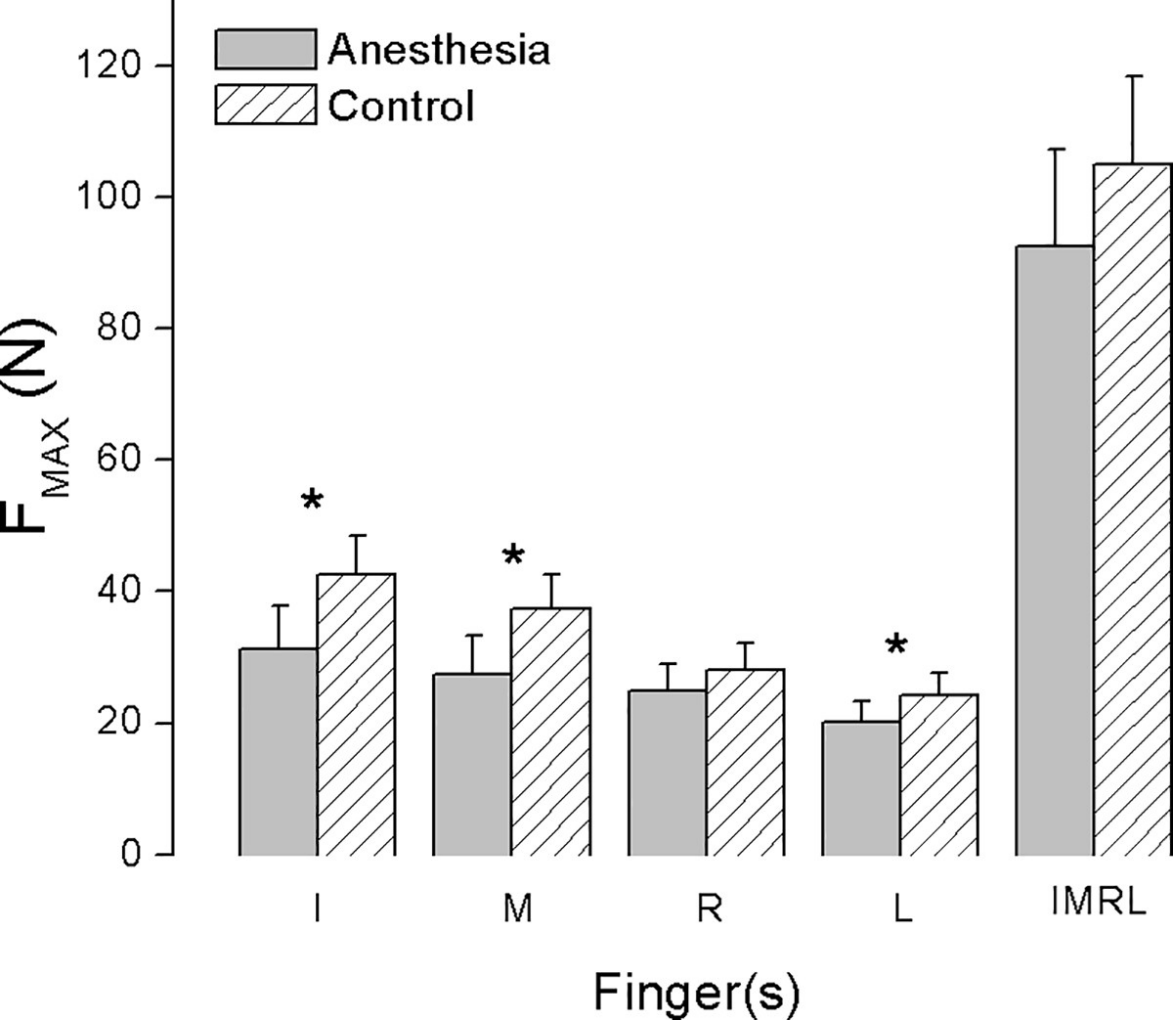
Averaged maximal pressing force in MVC task (mean ± SE). The maximal pressing force (F_MAX_) exerted by task-instructed fingers with I, M, R, L individually or IMRL combined, averaged across subjects in Anesthesia and control session separately. Asterisk indicates significant differences between sessions (P < 0.05).

We plotted the averaged F_MAX_ (mean ± standard error) by individual and then all fingers across subjects in both control and anesthesia sessions in Fig 2. While performing the voluntary maximal force contractions, subjects produced lower F_MAX_ after the selective digital anesthesia procedure (main effect of *Session*: F_[1,11]_ = 5.735, p < 0.001). In particular, subjects significantly reduced their maximal force production when using anesthetized fingers (I and M) as well as the non-anesthetized little finger. When using all four fingers, however, the total F_MAX_ observed during anesthesia and control sessions were not significantly different from each other (interaction effect of *Session* × *Cond MVC* (F_[4,44]_ = 2.813; p < 0.01; post hot comparison tests showed significant difference between sessions for conditions of I, M, and L) S1 Table.

### Enslaving task

We presented the averaged enslaving matrix entries (*E*, calculated for each individual subject for anesthesia and control sessions separately) across all subjects in Table 1. As the master finger, the index finger showed the least enslaving forces by other fingers, while the ring and little fingers were the most enslaved fingers (see bold values in Table 1) (main effect of *Master Finger*: F_[3,33]_ = 376.461; p < 0.001), whereas we did not observe any alternation of enslaving matrix before and after digital anesthesia (no main or interaction effect due to *Session*) S2 Table.

**Table 1:** Averaged *E* calculated from enslaving task across subjects (MEAN±SE).

Master Finger Enslave Finger	Control Session				Anesthesia Session			
	I	M	R	L	I	M	R	L
I	**0.95±0.03**	0.03±0.01	0.01±0.00	0.03±0.01	**0.94±0.03**	0.03±0.01	0.02±0.01	0.04±0.01
M	0.05±0.02	**0.91±0.03**	0.05±0.01	0.01±0.00	0.11±0.03	**0.83±0.09**	0.11±0.07	0.01±0.00
R	0.04±0.02	0.12±0.02	**0.78±0.04**	0.07±0.01	0.02±0.01	0.14±0.03	**0.80±0.03**	0.07±0.02
L	0.05±0.01	0.02±0.01	0.14±0.02	**0.83±0.03**	0.05±0.02	0.02±0.01	0.22±0.04	**0.76±0.04**

Values in bold denote master finger relative contributions.

### Synergy task

**Fig 3:**
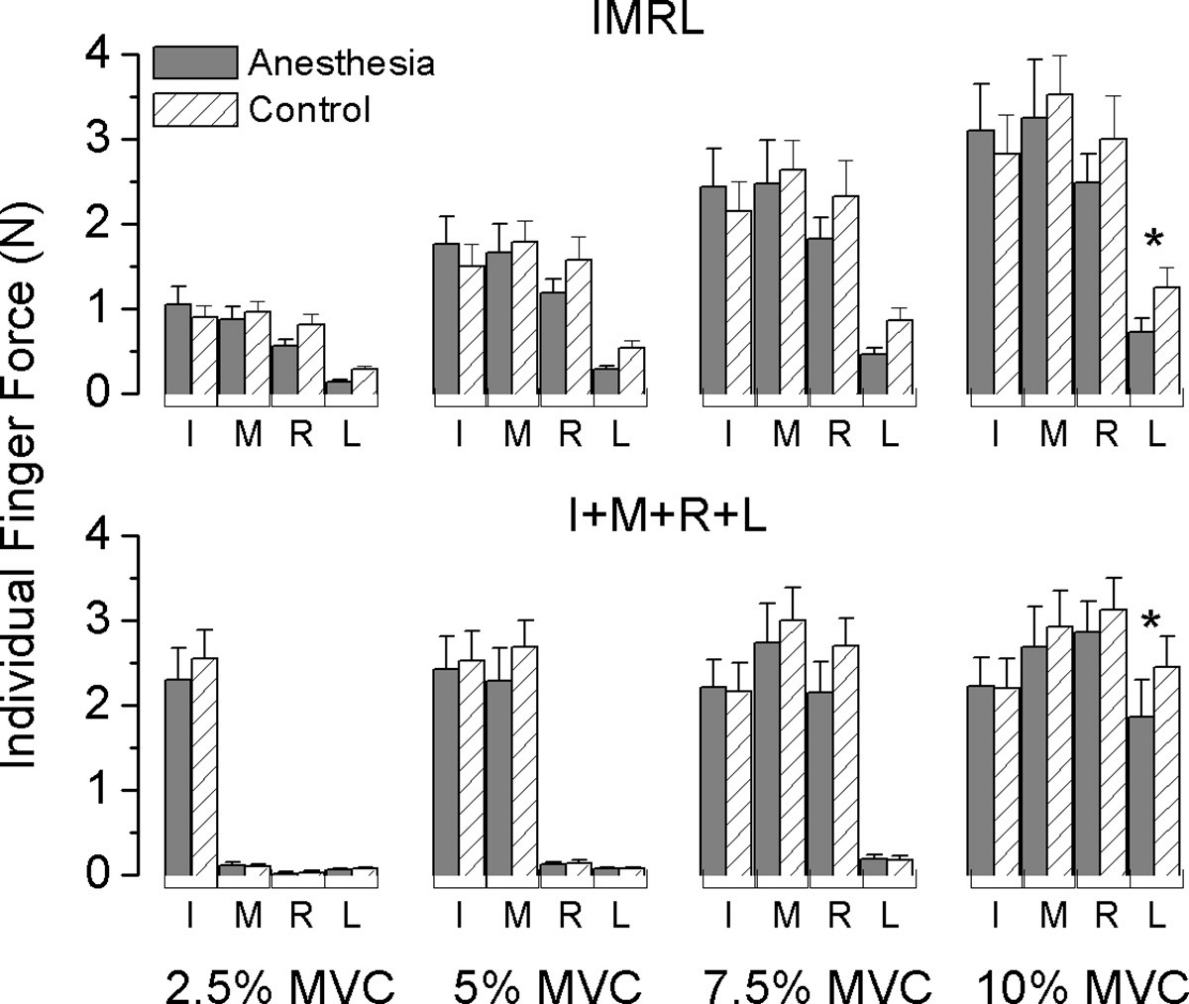
Averaged individual finger force in synergy tasks (mean ± SE). Absolute forces (in Newton) contributed by each individual finger as a function of task-required force levels (ranged from 2.5% - 10% MVC) are plotted for IMRL synergy task (top panel) and I+M+R+L synergy task (bottom panel) respectively. Plotted forces are firstly averaged over each force level per trial, task and subject and further averaged across subjects in Anesthesia and control session separately. Asterisk indicates an interesting significant difference between sessions at a non-local digit L (P < 0.05).

In Fig 3, we plotted the averaged individual finger forces from all subjects in both sessions for task IMRL and I+M+R+L separately. Subjects were asked to trace force template target line by using all four fingers together throughout the IMRL task; in the I+M+R+L task, subjects started with only I producing 2.5% MVC and added the next finger for each subsequent force level. Digits’ involvement as shown in Fig 3 confirmed that subjects performed the tasks as instructed, that is non-instructed fingers (MRL, RL, and L at force levels of 2.5%, 5%, and 7.5% MVC, respectively) barely produced forces during the task of I+M+R+L, yet all four fingers significantly contributed to the total force in the IMRL task. At the 10% MVC force level (highest), both tasks required all four fingers’ involvement. In this scenario, L was the least loaded finger among the four (main effect of digit: F_[3,33]_ = 13.538; p < 0.001; posthoc comparison tests showed that force produced by L was significantly lower than I, M, and R, all p < 0.05). However, there was a discrepancy in total force distribution among the digits between I+M+R+L and IMRL tasks in which four fingers shared the total force in an even fashion in the I+M+R+L task but not in IMRL. For example, R and L showed almost equal contribution (Anesthesia: 49%; Control: 52%) to the total force when compared with I and M in the I+M+R+L task but showed much less contribution (Anesthesia: 34%; Control: 40%) in the IMRL task (interaction effect of *Cond Synergy × Digit*: F_[3,33]_ = 7.481, p < 0.005; posthoc comparison tests showed that in IMRL, the force produced by L was significantly lower than I, M, and R, and in I+M+R+L, the force produced by M was significantly higher than I and L, all p < 0.05). Subjects reduced their L finger contribution from control to anesthesia session (I+M+R+L: from 23% to 19%; IMRL: from 12% to 8%) but retained force contributions from others in both sessions (interaction effect of *Session × Digit*: F_[3,33]_ = 3.38, p < 0.05; posthoc comparison tests showed that L produced a significantly lower force in anesthesia than in control session, yet no significant difference was found between two sessions for other digits).

**Fig 4:**
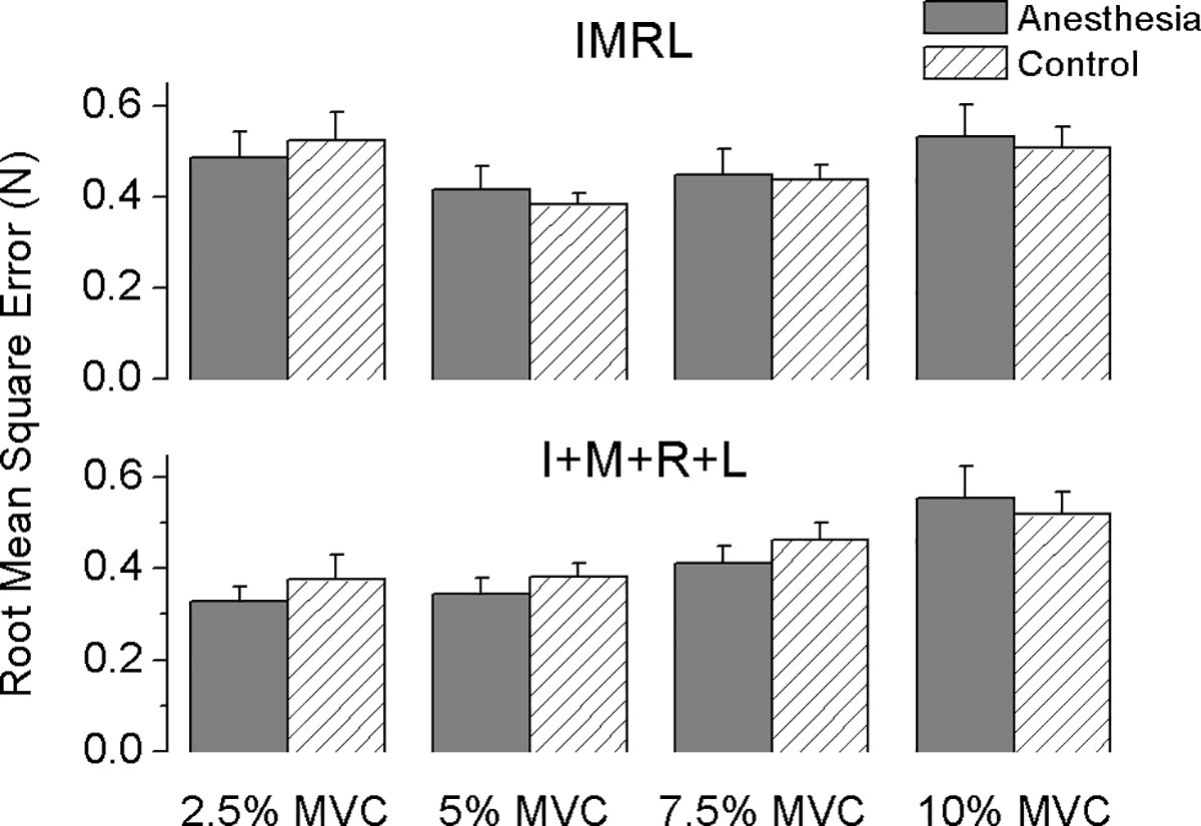
Averaged root mean square error in synergy tasks (mean ± SE). Root mean square error (RMSE) by task-instructed finger(s) as a function of task-required force levels are plotted for IMRL synergy task (top panel) and I+M+R+L synergy task (bottom panel) respectively. Plotted RMSE are firstly averaged over each task-required force level and further averaged across subjects in Anesthesia and control session separately. Note relative interaction effects were not directly applied on the plot (an asterisk in usual).

To quantify subjects’ actual force performance relative to the task-required force, we plotted the average RMSE across subjects at different force levels in both sessions for IMRL and I+M+R+L tasks separately in Fig 4. In general, subjects presented larger errors when using more digits than only a few. Higher RMSE values were observed in the IMRL rather than the I+M+R+L tasks (main effect of *Cond Synergy*: F_[1,11]_ = 5.663; p < 0.05) and subjects presented higher error values when using three and four digits (7.5% and 10% MVC) than one and two digits (2.5% and 5% MVC) in the I+M+R+L task (interaction effect of *Force Level × Cond Synergy*: F_[3,33]_ = 11.805, p < 0.001; posthoc comparison tests showed that in I+M+R+L task, RMSE values at two lower force levels were significantly lower than that at two higher force levels, whereas in the IMRL task, RMSE values at 2.5% and 10% MVC force levels were higher than the other two, all p < 0.05). This performance discrepancy between IMRL and I+M+R+L tasks was present only during anesthesia session; subjects in the control session presented similar errors in both synergy tasks (interaction effect of *Session×Cond Synergy*: F_[1,11]_ = 4.658; p < 0.05).

**Fig 5:**
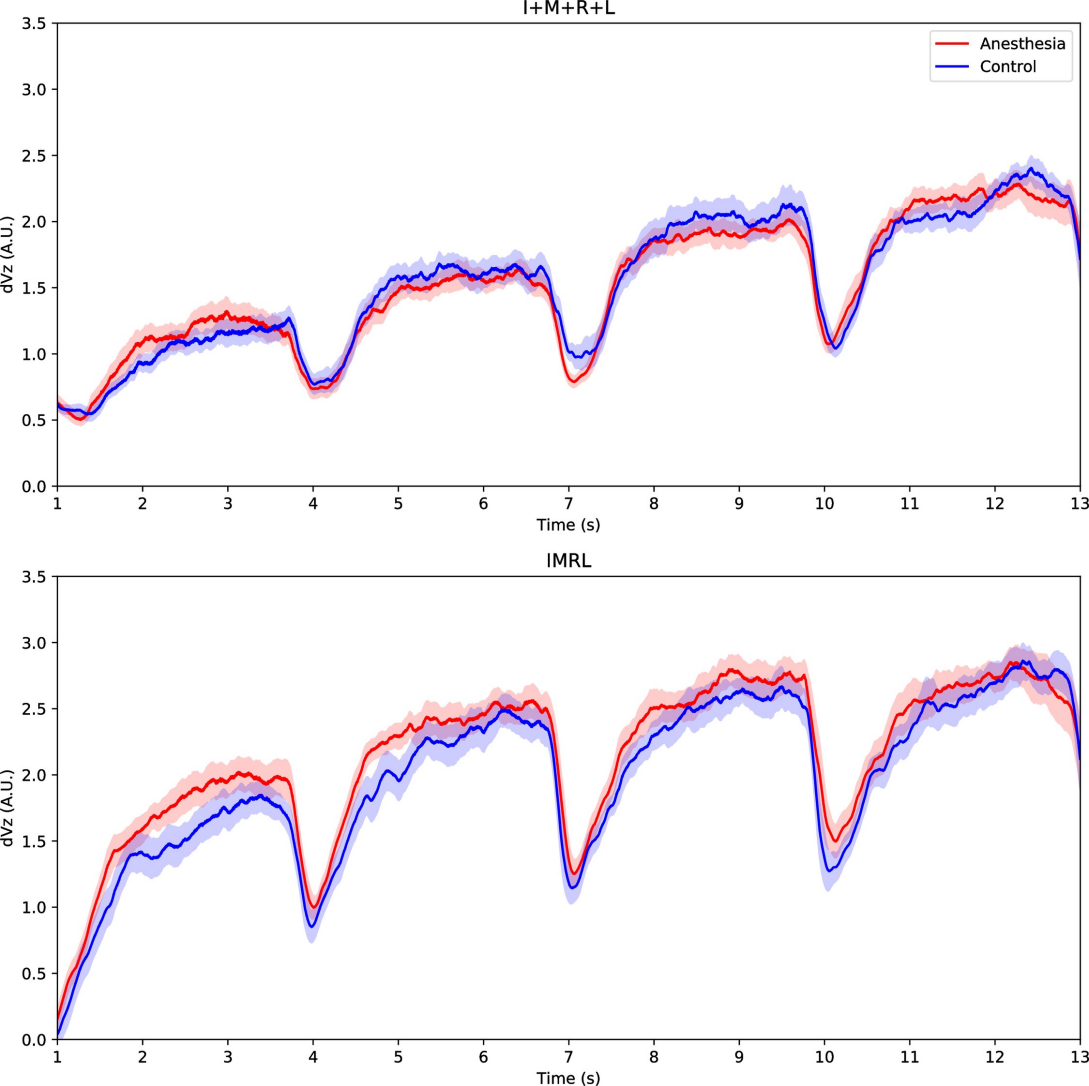
Averaged time profiles of force-stabilizing synergy indices in synergy tasks. The synergy index (ΔV_Z_) quantifying multi-finger coordination of total force output is calculated in four-dimensional space for both I+M+R+L (top panel) and IMRL synergy tasks (bottom panel) respectively. Plotted ΔV_Z_ values are firstly z-transformed, and further averaged across subjects in the anesthesia (red solid line with standard error) and control session (blue solid line with standard error).

In Fig 5, we plotted the average time profiles of ΔV_Z_ indices across subjects during anesthesia and control sessions for the IMRL and I+M+R+L synergy tasks. These indices were relatively high in both synergy tasks and transiently decreased when subjects moved from one force level to the next. Values of ΔV_Z_ increased as the instructed force production increased, corresponding to more involved fingers in the I+M+R+L task but not the IMRL task, as indicated by a robust main effect of *Force Level* (F_3,33_ = 201.04; P < 0.001). There was also a marginal *Force Level* × *Cond Synergy* interaction (F_3,33_ = 2.873; P = 0.051) arising from differential effects of adding fingers versus additional force production: in the I+M+R+L task, ΔV_Z_ increased relatively linearly as fingers were added, whereas ΔV_Z_ plateaued as force level increased above 5% MVC in the IMRL task. ΔV_Z_ was also higher in the IMRL task than the I+M+R+L task (main effect of *Cond Synergy*: F_1,11_ = 57.18; P < 0.001) across force levels. We observed no main effects of anesthesia (F_1,11_ = 3.17; P > 0.1).

ΔV_Z_ summarizes the relative amount of V_UCM_ (across-trials variance which does not affect task performance) and V_ORT_ (across-trials variance in task performance). ΔV_Z_ increased as force production increased because V_UCM_ increased (main effect of *Force Level*: F_3,33_ = 28.11; P < 0.001, with V_UCM_ at each successive force level larger than the previous one) while V_ORT_ did not increase as much (the main effect of *Force Level* was significant F_3,33_ = 12.33; P < 0.001 but post hoc tests showed only that V_ORT_ at 10% was significantly larger than the other force levels). Similarly, V_UCM_ was higher in IMRL than I+M+R+L (F_1,11_ = 14.36; P = 0.003) across force levels, while V_ORT_ was lower in IMRL than I+M+R+L at the 2.5% force level (where only one finger was instructed to press), but V_ORT_ was similar between tasks at the other force levels (*Cond Synergy* × *Force Level* interaction: F_3,33_ = 5.74; P = 0.003). While V_UCM_ was not significantly modulated by anesthesia, V_ORT_ was generally lower during anesthesia than control sessions (main effect of *Cond Synergy*: F_1,11_ = 6.357; P = 0.028), although it was only significantly lower under during anesthesia than control sessions at 2.5% and 10% force levels (*Session* × *Force Level* interaction: *F*_3,33_ = 3.05; *P* = 0.042) S3 Table.

## Discussion

In the present study, we examined the effects of selective digital deafferentation on multi-finger synergies during isometric pressing as a function of total force requirement and explicit involvement of different numbers of digits. Our results quantified these effects from three aspects: (1) maximal force ability; (2) force-tracing performance; and (3) multi-finger synergies. In the introduction, we formulated two hypotheses regarding the effect of selective digital anesthesia on multi-finger synergies: first, that anesthesia would result in decreased indices of synergy, and second that this decrease would be more evident at higher levels of force production. Neither of these hypotheses, however, were supported by our results: synergies did not decrease under selective anesthesia, and we did not observe differences between anesthesia and control sessions to depend on force production level. We further discuss the roles that sensory information played in these results and interpret our findings in context of relevant literature.

### Somatosensory contributions to maximal force abilities

The magnitude of voluntary force development relies on multiple factors, including motor unit recruitment and motor unit discharge rates [39–40]. We found decreased maximal force capacity after anesthesia, which agrees with previous findings [14,32,35] (Fig 2). These results construct a straightforward relation between maximal force ability and sensory-based contributions. Maximal voluntary force tasks require fast contractions (such as ramp contraction [41]), yet presents no explicit force goal. For this reason, MVC tasks are often assumed to be feedforward and thus it is not clear why reduced sensory feedback would lead to decreased force production capacity. One possible role for peripheral sensory signals (feedback processes) in MVC tasks is protective, so loss of sensation could make the central nervous system (CNS) decrease force output as a cautionary measure so as not to injure the periphery. Others have suggested that deafferentation results in higher levels of co-contraction, resulting in lower net forces [42].

### Local sensory deficits and non-local motor effects

Deafferentation-induced motor deficiency was not limited to the anesthetized (local) digits. Instead, we observed ‘non-local’ effects similar to our findings in earlier experiments using the same selective deafferentation model [14,32]. The non-local effects observed in the present study include a significant decrease in MVC from the little finger after sensory removal from other digits (I and M) (Fig 2). Additionally, the little finger significantly decreased its share of the force when working with together with other fingers during the synergy tasks (Fig 3). This could be counterintuitive since one might assume that a digit with intact sensation would compensate for those with reduced sensation by producing more of the force. We have previously interpreted similar findings based on the idea that integrating information from anesthetized and intact digits presents a larger challenge for the CNS [14,43].

We think another line of evidence for this interpretation is visible in the RMSE results. We quantified subjects’ sub-maximal force performance based on how much force production deviated from the task-required force target; our findings showed performance is not necessarily dependent on the amount of force produced, but rather on digital involvement instead. That is, when all four fingers were involved (IMRL task), RMSE was similar across force levels. In contrast, when fewer fingers were instructed to press (I or IM), lower values of RMSE were observed (Fig 4). Further, the reduction in RMSE occurred during anesthesia but not during the control session, as indicated as an interaction effect between sessions and tasks. This is consistent with the idea task performance can be retained when only anesthetized digits are utilized, but coordinating digits with different sensory abilities presents a particular challenge. This hypothesis could be further tested by having participants perform the task in the opposite direction (begin pressing with L, then R, etc.) to disentangle the role of the index and middle fingers (which are stronger and less enslaved) from that of anesthesia in this effect. Nonetheless, the observation of anesthesia-related effects on both local and non-local motor outputs is consistent with the possibility that sensory information from individual digits may be shared among others [32].

### The role of somatosensory feedback in organizing multi-digit synergies

A major goal of the present study was to investigate the contribution of somatosensory information to the structure of inter-trial variance. In isometric pressing tasks, a synergic structure of variance appears to be closely related to the availability of visual feedback on the task variable, but in some cases other sensory modalities can play a role. For example, one study [16] altered subjects’ proprioception by applying vibration on the palm or wrist surfaces and indicated subjects’ motor synergy strength was decreased, although synergies were still present. Similarly, results from a recent prehension study [14] revealed a reduction in the synergy index in the absence of selective digital sensory feedback in grasping tasks after digital anesthesia. However, many studies have reported loss of synergic structure of variance in isometric pressing when visual feedback was removed [44–45], and Koh and colleagues [17] reported no change in cross-trial structure of variance following removal of somatosensory feedback from all task-involved digits.

We did not find that digital anesthesia weakened synergies in the present isometric pressing task. In fact, we found strong synergies in the absence of somatosensory feedback, similar to Koh’s report [17]. Note that both our paradigm and Koh’s provided explicit visual feedback that allowed prompt and precise error correction during the task. This result suggests, in agreement with previous studies, that synergic structure of variance can be easily organized with visual feedback alone. Another piece of evidence for this interpretation is our finding of synergies even in the 2.5% force level of the I+M+R+L task: an instance when theoretically no synergy should be observed. However, our results suggest that merely showing visual feedback from all fingers–even when participants are explicitly instructed to press with only one finger–is enough to induce a synergic structure of across-trials variance. These findings corroborate previous studies investigating the effect of adding digits to isometric pressing [44–45] which also reported minimally altered structure of variance as additional digits were added to an isometric pressing task and synergic structure of variance when instructed to press with the index finger only.

Even if somatosensory feedback is not sufficient for organizing multi-digit synergies during isometric pressing tasks, it appears to play a role in motor variance. In particular, we observed decreases in V_ORT_ under anesthesia, indicating that somatosensory feedback actually drive small fluctuations in task performance (similar to the lower RMSE observed under anesthesia). High indices of synergy are often interpreted as healthy (especially because some neurological populations display lower indices of synergy), therefore, our observation of increased ΔV_Z_ during the anesthesia session may be a surprising outcome. In fact, increased indices of synergy can also be observed in highly decoupled systems which are joined only at a high feedback level. A good example of this phenomenon is the very large index of synergy observed in isometric tasks performed by two people with shared visual feedback [46] which occurs because, across trials, motor output from individual people are very high compared to when one person produces all of the output. However, in this case, visual feedback ensures that the task is performed at an acceptable level across trials (V_ORT_ is kept relatively low), leading to very high ΔV_Z_ values. Similarly, if the CNS has little access to forces produced by individual fingers, it may just find a solution that works on a given trial instead of further refining these individual force levels based on other criteria that are not explicitly involved in task completion–like comfort.

Dovetailing on the theme of somatosensory information moderating “communication” between digits, in our I+M+R+L task, ΔV_Z_ increased approximately linearly for anesthesia session. In contrast, the increase in ΔV_Z_ associated with adding digits saturated in the control session. These results could occur if the addition of digits is relatively independent under anesthesia (e.g. because the CNS does not have access to information regarding what other fingers are doing under anesthesia), resulting in relatively high variance across trials in the forces (modes) produced by individual fingers. In contrast, finger forces may be re-organized by the CNS as additional fingers are added when somatosensory function is intact, resulting in more stereotypical values of fingers forces across trials. These more stereotypical values could represent individual preferences about sharing force production between fingers.

Synergic structure of inter-trial variance could be a product of feedforward control [15, 18] where finger forces for a given trial are “selected” by the CNS from some distribution and implemented with motor noise, or feedback [8, 19] control processes where variability in output which do not affect task performance are disregarded. While the role of feedback in general is very important for organizing synergies, our results suggest that somatosensory information in particular might be used to optimize secondary, *implicit* objectives of the task like comfort. This can be seen as a stage in synergy learning [12] within the framework of the uncontrolled manifold hypothesis [8], where task performance is first ensured before the CNS settles on specific (preferred) levels of elemental output occur. Similarly, it could be explained in the parlance of optimal feedback control [18]: reduced availability of somatosensory information may alter the ability of the CNS to optimize a cost function that includes terms for individual finger forces (evaluated in terms of somatosensory information from cutaneous receptors), or change the cost function being evaluated to disregard such terms if they are known to be corrupt; however, given that the task is performed in terms of visual feedback, the CNS still preferentially stabilizes output which are consistent with task success.

## Conclusions

Temporary somatosensory deprivation via digital anesthesia decreases maximal voluntary contraction force, but it does not detrimentally affect target-tracing force performance or the organization of multi-finger motor synergies. Our study indicates that the CNS is capable of retaining the force-stabilizing synergies in a redundant system. However, there may be costs associated with coordinating both sensory-impaired and -intact motor elements. These results may be explained in the optimal feedback control context by assuming that reduced somatosensory input does not directly interfere with the CNS’ ability to execute an isometric task with redundant elements, but altered structure of variance may indicate that it interferes with the CNS’ ability to optimize secondary motor goals related to comfort or distribution of force to specific digits.

## Supporting information

S1 Table Subjects’ MVC forces. Data of individual subjects’ F_MAX_ calculated in each MVC conditions during anesthesia and control sessions.
(XLSX)

S2 Table Subjects’ enslaving matrix. Enslaving data of 4×4 *E* calculated for each individual subject in enslaving task during anesthesia and control sessions.
(XLSX)

S3 Table Subjects’ ΔVz. Individual subjects’ synergy indices data of ΔV_Z_ calculated as a function of time in synergy task of IMRL and I_M_R_L respectively, during anesthesia and control sessions.
(XLSX)
